# Dimethyl fumarate for ischemic stroke

**DOI:** 10.18632/oncotarget.15357

**Published:** 2017-02-15

**Authors:** Reiner Kunze

**Affiliations:** Institute of Physiology and Pathophysiology, Heidelberg University, Germany

**Keywords:** dimethyl fumarate, fumaric acid esters, Nrf2, HCAR2, HIF, Neuroscience

In a series of proof-of-concept experiments using *in vitro* and *in vivo* models of ischemic stroke, we provide evidence that dimethyl fumarate (DMF) strongly reduces neuronal cell death upon ischemic insult through pleiotropic anti-inflammatory and cytoprotective effects [1, 2]. DMF is a derivative of the fumaric acid, an intermediate product of the citric acid cycle. DMF is marketed as Tecfidera^®^ (formerly known as BG-12; Biogen, USA), and is used as a peroral treatment for relapsing-remitting multiple sclerosis (MS), an autoimmune inflammatory disease of the central nervous system (CNS) [3]. Notably, MS and ischemic stroke share several pathophysiological mechanisms including oxidative stress, and recruitment and pro-inflammatory activation of resident and circulating immunocompetent cells.

In mice suffering from focal ischemic stroke, orally administered DMF clearly reduced the development of vasogenic edema by maintaining the integrity of interendothelial tight junctions (TJ) which form a physical barrier restricting the paracellular influx of blood-derived molecules and fluid into the CNS [1]. We further demonstrated that DMF diminished the pro-inflammatory activation of astrocytes and brain microvascular endothelial cells in response to ischemic stress *in vitro* and suppressed monocyte migration across a brain endothelial cell monolayer presumably by downregulation of leukocyte adhesion molecules [1]. Using murine organotypic hippocampal slice cultures and neuronal cell lines subjected to ischemia-reoxygenation *in vitro*, we then showed that DMF ameliorates neuronal viability also through direct cytoprotective actions independent of its effects on non-neuronal brain cells [2].

Pharmacokinetic studies in human volunteers indicate that orally applied DMF is rapidly cleaved in the alkaline environment of the intestine into monomethyl fumarate (MMF) by esterases, and additionally undergoes a strong first-pass effect in the liver. Thus, MMF as the main metabolite is considered the active compound in the body [3]. Hence, we compared the neuroprotective potential of both fumaric acid esters (FAE), and confirmed equal ischemic tolerance of neurons treated either with DMF or MMF [2]. Although the exact mechanism of action of DMF in MS patients is still unclear, a number of experimental animal studies have provided important insights into molecular mechanisms which might underlie the favorable effects of oral DMF [4-7]. FAE are electrophiles and serve as “Michael acceptors” that covalently link to nucleophilic thiol groups on macromolecules, as it has been shown for reactive cysteine residues of the Kelch-like ECH-associated protein 1 (Keap1) [4, 5]. Keap1 acts as an inhibitor of nuclear factor (erythroid-derived 2)-like 2 (Nrf2), a major transcription factor that orchestrates the expression of antioxidant, anti-inflammatory, and cytoprotective genes in response to electrophilic and oxidative stress conditions. S-alkylation of Keap1 causes the release of Nrf2 from Keap1, permitting its escape from proteosomal degradation, nuclear translocation and induction of gene transcription [3, 4]. In fact, we and others have demonstrated that blood circulating FAE can pass the blood-brain barrier, and activate the Nrf2 pathway in the CNS [1, 4]. Moreover, DMF-induced restoration of TJ proteins in endothelial cells, and ischemic tolerance of neurons were completely abolished in the absence of Nrf2 [1, 2]. Accordingly, in wild-type (WT) mice subjected to chronic experimental autoimmune encephalomyelitis (EAE), a common animal model of MS, delayed oral DMF treatment ameliorated the clinical course, improved axon preservation, and reduced astrocyte activation, while no clinical benefit was found in Nrf2 deficient mice [4]. In contrast, when starting oral DMF therapy parallel to EAE induction, DMF protected WT and Nrf2 deficient mice equally well from development of clinical and histologic features of acute EAE [5]. The benefit of DMF treatment in both Nrf2 deficient and WT mice was associated with reduced frequencies of Th1 and Th17 cells, and enhanced induction of anti-inflammatory M2 monocytes [5]. These observations suggest that anti-inflammatory and immunomodulatory responses initiated by DMF may also occur through alternative pathways, independent of Nrf2. In this regard, Chen et al. reported that favorable effects of oral DMF treatment in acute EAE were partially abolished in mice deficient for the hydroxycarboxylic acid receptor 2 (HCAR2) [6], a G_i_-coupled cell surface receptor known to suppress pro-inflammatory activation and chemotactic recruitment of immune cells such as dendritic cells and macrophages. Mechanistically, MMF has been shown to impede infiltration of neutrophils into the CNS during acute EAE in an HCAR2-dependent, but Nrf2-independent, manner [6]. Accordingly, Parodi et al. demonstrated that MMF activates the AMPK/SIRT1 axis in microglia through HCAR2, resulting in inhibition of NF-κB-dependent release of pro-inflammatory molecules [7]. Future investigations will show whether HCAR2-dependent anti-inflammatory mechanisms in microglia and infiltrating blood-derived leukocytes contribute to FAE mediated CNS protection in stroke. Finally, a previous report raised the possibility that FAE might activate hypoxia-inducible transcription factors (HIFs) through inhibition of prolyl-4-hydroxylase domain (PHD) proteins by competing for their natural co-substrate 2-oxoglutarate [8]. However, our recent data did not confirm that either DMF or MMF activate the HIF pathway in various brain cells in a substantial way suggesting that HIF-dependent cytoprotective mediators may not significantly contribute to FAE mediated neuroprotection within the pathophysiological context of stroke [2].

Although our findings point to a therapeutic benefit of DMF in acute ischemic stroke, future studies addressing therapeutic time window, dose-response relationship and drug side effects are indispensable for a serious evaluation of the therapeutic potential of DMF. The hope remains that oral DMF treatment with its exceptional clinical efficacy and well-established safety profile in humans might be the first drug therapy overcoming the translational roadblock that plagues the search for an effective treatment for acute human stroke during the recent decades.
